# Towards valorizing natural coals in sodium-ion batteries: impact of coal rank on energy storage

**DOI:** 10.1038/s41598-020-72759-0

**Published:** 2020-09-28

**Authors:** John Abou-Rjeily, Noureddine Ait Laziz, Cecile Autret-Lambert, Abdelkader Outzourhit, Moulay-Tahar Sougrati, Fouad Ghamouss

**Affiliations:** 1 Laboratory of Physical-Chemistry of Materials and Electrolytes for Energy (PCM2E), University of Tours, Tours, France; 2 Laboratory of Physics for Solid and Thin Films (LPICM), University of Cadi Ayyad, Marrakech, Morocco; 3 Materials Research Group, Microelectronics, Acoustics and Nanotechnologies (GREMAN), University of Tours, Tours, France; 4 Charles Gerhardt Institute (ICGM), CNRS UMR 5253, University of Montpellier, Montpellier, France; 5 Department of Materials Science, Energy, and Nano-engineering, Mohamed VI Polytechnic University, Ben Guerir, Morocco

**Keywords:** Chemistry, Energy science and technology, Materials science

## Abstract

Coal samples of different ranks were investigated through various compositional, morphological/structural, and textural experiments prior to their electrochemical implementation in Na-ion half-cells. The purity of coals proved insignificant while distinctions in the flake size, pore width, pore distribution, I_D_/I_G_ ratio, crystallite parameters (L_a_ and L_c_) along with adjacent parameters, such as the R-empirical parameter, i.e., limited parallel graphene stacking proved more relevant for Na^+^ storage into the negative host electrodes. Coal powders were identified via a two-step TGA analysis technique displaying the overall carbon content of the coals and the impurities. Coal-based anode materials were prepared from raw and pyrolyzed coals (at 800 °C under argon gas-flow) and cycled in Na-ion half-cells to further investigate the impact of the coal rank on the energetic properties. High volatile bituminous coal with lower graphene stacking and augmented nanoscopic pores delivered higher reversible capacity in comparison with semi-anthracite coal, whether in their raw (67 vs. 54 mAh/g) or pyrolyzed (214 vs. 64 mAh/g) states, respectively vs. Na/Na^+^. The dominance of HVBC over SAC due to enhanced properties as R-empirical parameter, I_D_/I_G_ ratio, and internal porosity. This study provides an exhaustive methodology to assess other carbonaceous anode materials further to evaluate their energy storage capabilities.

## Introduction

Rechargeable sodium-ion batteries (NIBs) with reliable performance have significant advantages over lithium-ion batteries (LIBs) due to price reductions in the raw materials implemented in these systems^1,2^. The advantage of using low-cost coals in NIBs supplements further cost-assurances due to the abundance of these materials. Early studies in NIBs date back to 1980 when a primary Na^+^ electrochemical insertion in Na-TiS_2_ was effective^3^_._ However, failure in preventing non-destructive reversible intercalation into graphite led to abandoning this technology due to the absence of suitable negative hosts for reversible Na^+^ intercalation^4,5^. Consequently, the "house of card" model for Na^+^ storage proposed by Dahn et al. triggered an academic interest in developing Na-ion cells, increasing the number of publications tremendously during the last decade with the availability of suitable anodes^2,4,6^. NIBs deliver great cost cut-backs; nevertheless, challenges such as lower operating voltage, increased atomic weight leading to decreased gravimetric energy density, and higher equilibrium potential in aqueous solutions are reported^7^. Nonetheless, implementing this technology in large-scale stationary energy storage is promising, particularly in renewable energy collection hence providing economic and ecological assurances^8,9^.

Preparing high capacity coal-based anodes for energy storage was reported in lithium-ion batteries (LIBs) by Dahn et al.^10^. Calcination of eight different coal samples at 1000 °C and other carbon sources at 900–1100 °C led to the formation of limited parallel graphene stacking (i.e., R empirical parameter). By turn, this augments the number of nanoscopic pores leading to increased reversible lithium intercalation that expands the number of nanoscopic pores leading to increased reversible lithium intercalation^10,11^. This group has remarked a tendency amid the parallel graphene stacking and the obtained specific capacity, and as this value increases, the specific capacity decreases. The impact of coal rank was marked; high volatile bituminous coal delivered the best reversible capacity (450 mAh/g vs. Li) although they are not the highest in purity in comparison to other coals^10^. Coals of higher purity and increased carbon content, such as anthracite calcined at 1000–1150 °C, delivered a lower reversible capacity (370 mAh/g vs. Li)^12^. Meanwhile, coals of lower purity and decreased carbon-content such as lignite pyrolyzed at 1000 °C attained the lowest reversible capacity (340 mAh/g) and irreversible capacity (80 mAh/g). Pyrolysis of carbonaceous materials has proved to be an effective tool to enhance its properties. Nevertheless, other techniques such as nitrogen doping^13,14^ and tailoring in structure and designs^15^ are also useful techniques to improve the electrochemical property of the host materials.

Nevertheless, reports concerning coal-based anode materials implemented in sodium-ion batteries (NIBs) are scarce. Our group recently reported the use of high volatile bituminous coal as anode materials in NIBs and the impact of thermal treatment between 800 and 1200 °C. The highest reported reversible capacity attained was 223 mAh/g vs. Na and 409 mAh/g vs. Li for samples calcined at 800 °C^16^. Another sole study reports high purity anthracite pyrolyzed between 1000 and 1400 °C, delivering a reversible specific capacity of 222 mAh/g vs. Na^17^. Reports regarding lower purity coals (lignite) and the impact of coal rank on reversible Na^+^ intercalation were never reported. The environmental factors involved during coalification delivers different coal ranks^18^. In turn, each coal rank bases different compositions (purity), morphology (flake size and graphene-sheet ordering), structure (L_c_, L_a_, d_002_, R), and porosity. The availability of different coals of various ranks suggests that each coal rank has a separate energetic property due to different intrinsic properties, a matter discussed in this work comparing two coals of distinct ranks.

## Methods

Natural coal samples collected from "Jerada" abandoned mine in Morocco were disregarded as mining waste materials since they are considered economic burdens. These materials imposed environmental risks along, causing several fatalities due to collapsing mines in 2018. Thermo-gravimetric analysis (TGA) performed using a TGA 800 PerkinElmer on both coal samples under two different gas flows (nitrogen and air) at a 5 °C/min step in a ceramic substrate. This experiment permitted the differentiation amid volatile matter, fixed carbon, and the ash content of each coal sample leading to their rank determination. Elemental analyses were achieved by flash combustion and gas analysis techniques via a microanalysis elemental analysis service. Scanning electron microscopy (SEM) imagery was collected using a "Zeis ULTRA Plus" electron microscope, which was coupled with energy-dispersive X-ray spectroscopy (EDS) at altered magnifications and using different detectors, namely in-lens detector secondary electrons (SE1) (Everhart Thornley) type detector and angle-sensitive backscatter detectors (AsB) (4 quadrant detector). Using AsB provided images with distinct contrast and grey-scale associated with the atomic number (the bright regions have higher atomic number Z). The Z-contrast demonstrates channeling contrast (crystallographic and strain information); hence impurities (elements different from carbon) show a lighter/brighter coloring. EDS allows a qualitative and semi-quantitative analysis of the elements involved. Raman tests were carried out by using LabRam HR Evolution (Horiba) spectrometer. Powders were mounted on a glass lens and subjected to a green laser of 532 nm wavelength at a magnification of × 50 using a laser intensity of 10%. X-ray diffraction (XRD) using an X-ray diffractometer operating at 40 kV and 40 mA using Ni-filtered Cu Kα radiation (k = 0.15406 nm). XRD structural analysis using Bragg's law and Scherrer's equations permitted the calculation of the lateral crystallite size (L_a_), crystallite stacking height (L_c_), the interlayer spacing of the crystalline structure (d_002_), the average number of layers per carbon crystallite (N_average_), the average number of carbon atoms per aromatic lamellae (n). As reported by Dahn et al., the R-empirical parameter defining the ratio of the peak count rate at the [002] peak divided by the background level (estimated by linear extrapolation) at the same angle was calculated. Before these calculations, the contribution of the Al_2_O_3_ stub used was calculated and subtracted.

Characterizing the textural properties of the powders was done through the N_2_ adsorption method using a QUADRASORB EVO apparatus. Quenched solid-state functional theory (QSDFT) for cylinders and spheres geometry facilitated the calculation of pore-size distribution, cumulative pore volume, and pore width. Moreover, Brunauer–Emmett–Teller (BET) model was applied, since its theory serves in the measurement of the specific surface area of the materials. Samples were dried under vacuum at 200 °C for 3 h, and analysis under nitrogen adsorption/desorption at 77 K was carried out.

Two coal samples collected were hand crushed, ball-milled at 200 RPM for 1 h with acetone separately in agate jars with five 1 cm diameter agate balls. After drying in open air overnight, the active materials were further dried in a 110 °C oven. The thermal treatment of the powders was carried out at 800 °C under argon gas flow. Subsequently, slurries were prepared with 80 wt% of the active materials being mixed with carbon black (10 wt%), polyvinylidene fluoride (PVdF) (10 wt%) binder, and *N*-methyl-2-pyrrolidone (NMP) solvent. The prepared slurries were mixed overnight for enhanced dispersion and then deposited on aluminum foils using a semi-automatic machine (Elcometer 4340) with a desired 40 µm thickness (wet state). The electrodes were then left to dry in open-air overnight and then dried up for 4 h at 80 °C under reduced pressure. The electrodes were then cut into 0.9 cm diameter circles and introduced into an argon-filled glove box for cell assembly. The overall mass loading of the materials on the electrodes was around 2.0 mg/cm^2^.

Na-half cells were assembled in a Swagelok type cell with the prepared electrodes serving as the working electrode coupled with the Na-metal reference electrode. The electrodes were separated by an ionic conductive glass microfiber sheet (Whattman Grade GF/C) that allows the stocking of the electrolyte while preventing contact between the electrodes. The electrolyte of choice was a mixture of 1 M sodium hexafluorophosphate (NaPF_6_) dissolved in propylene carbonate (PC) and fluoroethylene carbonate (FEC) (95 to 5 ratios by weight). The latter is an electrolyte additive aiding in the formation of solid-layer interphase (SEI) in NIBs^6^. The electrochemical performance of Na-ion half cells was assessed via cyclic voltammetry (CV) and galvanostatic cycling with potential limitations (GCPL). These cells were discharged to 5 mV rather than 0 V to avoid unanticipated problems. Cyclic voltammetry was performed at a scan rate of ± 0.025 mV/s starting from the open-circuit voltage to 5 mV vs. Na.

## Results and discussion

**Figure 1:**
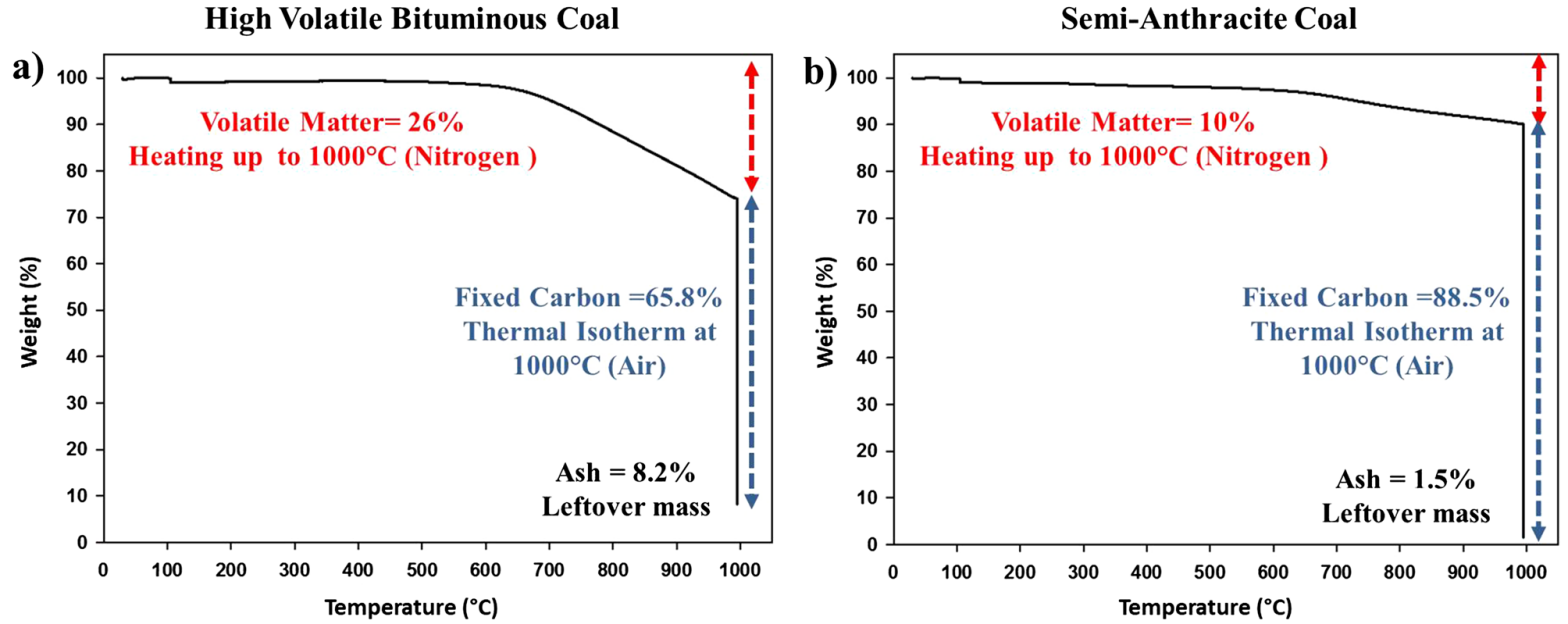
TGA curves presenting the weight variation as a function of temperature for: (**a**) High volatile bituminous coal and (**b**) Semi-anthracite coal.

Coal rank determination, as discussed in the experimental section, was carried out by a two-stage TGA experiment. The first stage promotes the identification of the water and volatile matter contents by heating the coal samples to 1000 °C under a nitrogen gas flow. The volatile matter is a group of combustible elements that vaporize when the sample is heated, and they are mainly methane, hydrocarbons, hydrogen and carbon monoxide, and incombustible gases (such as CO_2_). The second stage promotes the determination of the fixed carbon, i.e., the carbon in its free state and not combined with volatile materials^19^. Switching the gas flow into an airflow rather than nitrogen flow promotes the combustion of these materials leaving behind ash or non-combustible materials that are considered impurities. Figure 1 displays the differing composition of each coal. The first coal sample was identified as high volatile bituminous coal (HVBC), as reported in the literature^20,21^. On the other hand, the second coal sample displayed a different TGA profile with decreased volatile matter content, increased fixed carbon content, and minute ash content. The coal sample was identified as semi-anthracite coal (SAC). The disparity in the fixed carbon content that is caused by varying volatile matter and ash content is the main factor in determining the coals' ranks. HVBC has elevated volatile matter (26%) and ash content (8.2%) along with decreased fixed carbon content (65.8%) in comparison to SAC that contains an inferior volatile matter (10%) and ash content (1.5%) along with increased fixed carbon (88.5%).

The elemental analysis, as displayed in Table 1, indicates an increased carbon mass in HVBC in comparison to SAC. Nevertheless, we recognized that the latter is of a lower volatile nature (mostly of carbon nature); hence, the recorded carbon composition is confusing, and elemental analysis can prove troublesome for coal rank determination. Nevertheless, elemental analysis clarified the increased presence of sulfur impurities in HVBC than in SAC due to increased impurity level (suspected pyrite content). Furthermore, the elemental analysis identified the impurity nature in each sample, whereas TGA provided more accurate carbon content values allowing the determination of the two different coal ranks^16^.

**Table 1:** Elemental analysis of high volatile bituminous and semi-anthracite coals.

Coal sample	Carbon	Hydrogen	Nitrogen	Oxygen	Sulfur
HVBC	86.49	2.38	0.89	3.53	3.03
SAC	79.24	1.97	0.85	7.49	0.91

**Figure 2:**
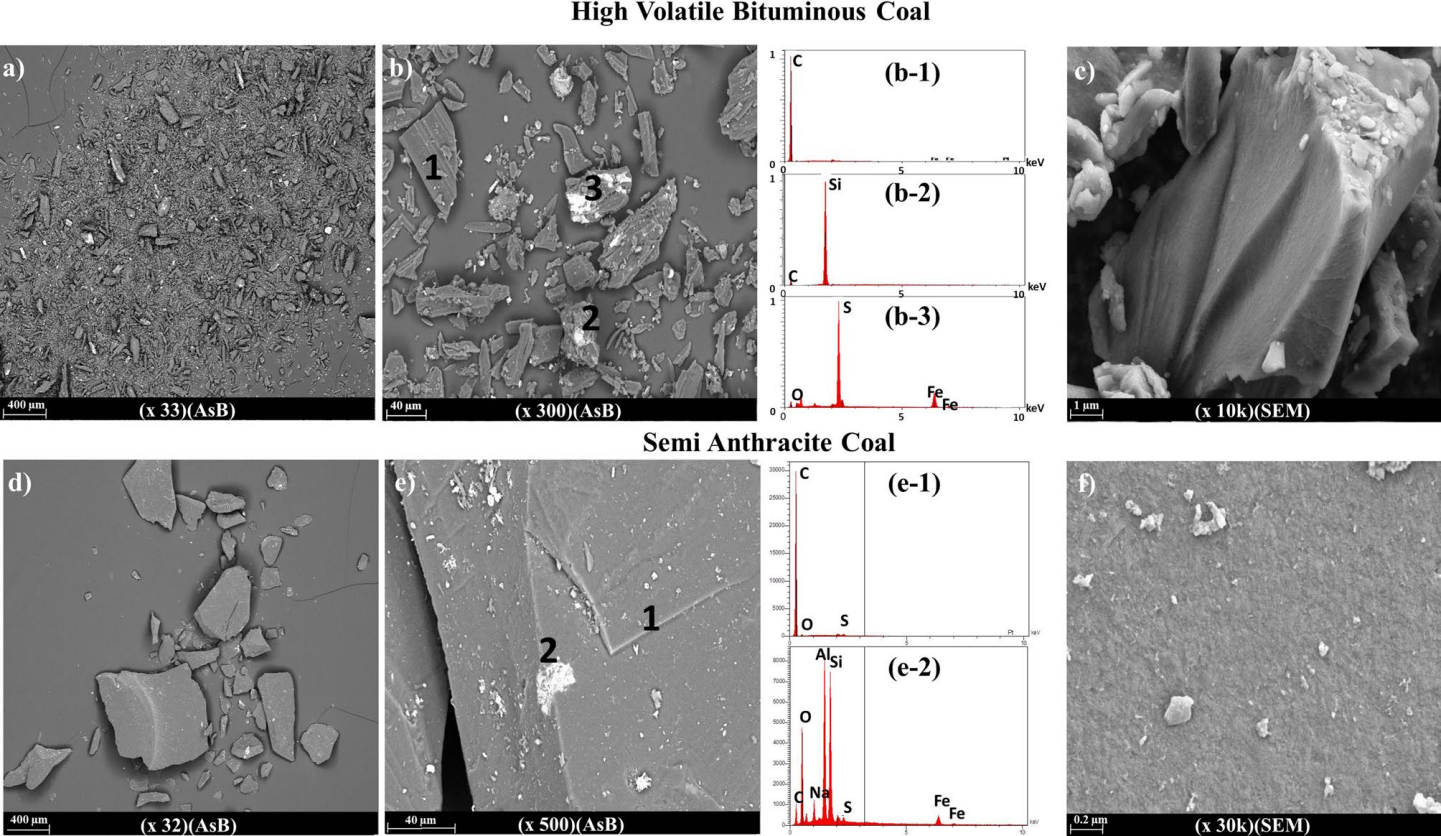
AsB imagery of HVBC at various magnifications (**a**) × 33 and (**b**) × 300 and SAC coal at various magnifications (**d**) × 32 and (**e**) × 300. EDS spot analyses of HVBC (**b**: 1–2–3) and SAC (**d**: 1–2). SEM imagery at high magnifications for (**c**) HVBC and (**f**) SAC.

Surveying the morphological and structural properties of these coals with low magnification via SEM images showed several differences between HVBC (Fig. 2a) and SAC (Fig. 2d) and at high magnifications (Fig. 2c,f). Different flake size distributions were mainly attributed to the nature of the raw materials since ball-milling, and crushing parameters were the same for both samples. However, even after ball milling, the flake size of the higher-ranked coal was majorly more full-grown and could develop energetic constraints. Although the two coals were obtained from the same mine, angle-sensitive backscatter (AsB) displaying a grey-scale contrast shows a higher presence and distribution of impurities in HVBC than in SAC. This observation is in coherence with previous TGA experiments where the ash content (mainly oxide impurities) was more evident in HVBC. Furthermore, energy-dispersive X-ray spectroscopy (EDS) spot analysis recognized the dark zones as carbon dominated regions for HVBC **(**Fig. 2b) SAC (Fig. 2e). Meanwhile, bright zones were identified as impurity saturated zones, mainly silicon-rich (Fig. 2b2) or sulfur and iron-rich (Fig. 2b3). Few spots contained a mixture of contaminants (Fig. 2e2) with additional impurities such as sodium, potassium, and aluminum, which are the typical composition of a natural rock. At higher magnifications, the SAC displays a nonfractured structure, whereas the HVBC acquires many fissures and cracks.

**Figure 3:**
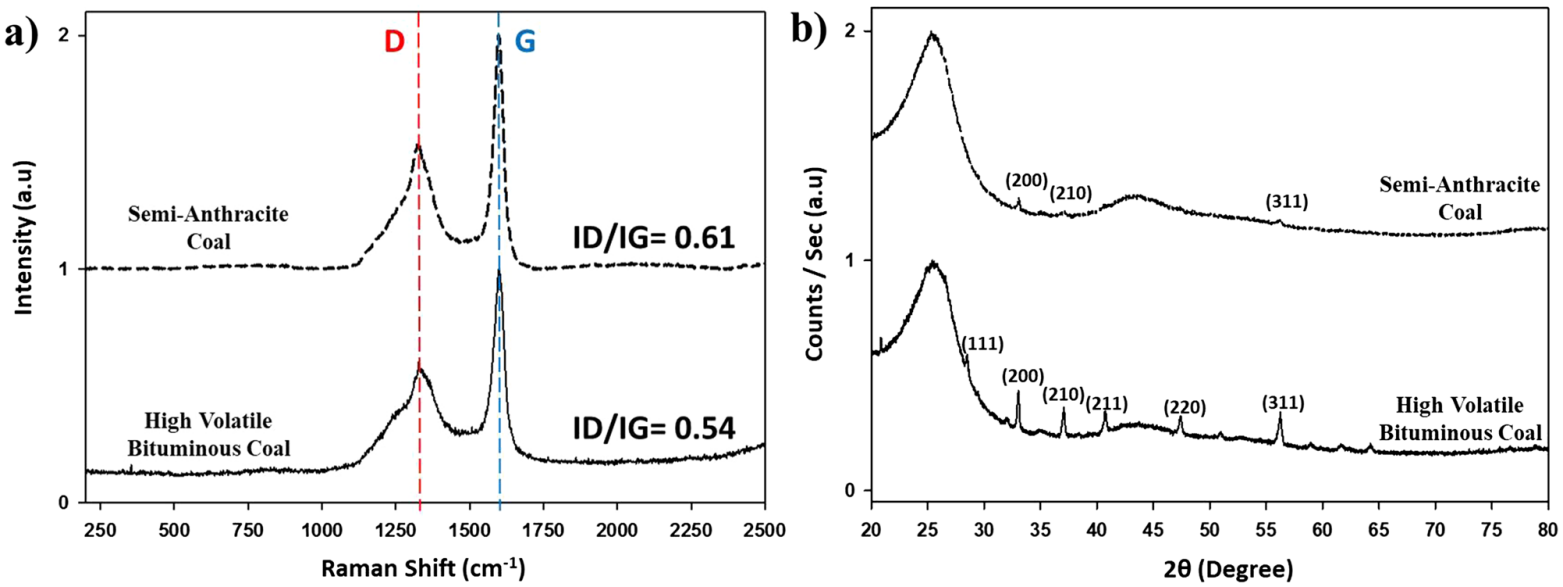
(**a**) Raman spectra and (**b**) XRD spectra for high volatile bituminous (black lines) and semi-anthracite coals (dashed black lines).

Higher ranked coals have higher carbon ordering due to increased pressure and thermal activity during their geological formation. The previous statement was proved correct by carrying out Raman spectra (Fig. 3a) and X-ray diffraction (Fig. 3b) tests. Analogous positions of the dispersive band (D) at around 1350 cm^−1^ and the graphitic band (G) at around 1582 cm^−1^ bands representing the A_1g_ symmetry and E_2g_ (both signify *sp*^2^ sites), respectively, characteristic of amorphous carbon were recorded. The first band shows the degree of carbon amorphicity, and the latter band shows the graphitic carbon criteria^22^. Comparing both coal samples displayed an increased I_D_/I_G_ ratio for the SAC, demonstrating higher carbon ordering in the structure of the coal. The augmentation of the I_D_/I_G_ in disordered carbonaceous material indicates ordering, unlike for graphitic carbonaceous materials, were the TK equation doesn't hold^22^. The development of the D band was also noticed when subjecting these coals to augmented heats. As the temperature increases, the ID/IG increases further, demonstrating the ordering of the materials^16,22^.

Meanwhile, in XRD, two analogous amorphous carbon peaks were evident between 20°–30° and 42°–45°. The first distinguishes the d_002_ peak, a representative of the interlayer spacing of the disordered carbons. The second peak represents an amorphous phase of carbon (hkl 100). The latter is more predominant and intense in the SAC coal since it is more ordered and since impurities restrain from interfering and augmenting the intensity of the peak thus, delivering coherent information that this coal is purer than HVBC. Furthermore, the pyritic phase was more identified in the lower rank coal with hkl contribution denoted in (Fig. 3b), whereas the higher rank coal bestows minute pyrite peaks. XRD structural analysis of the two raw coal samples, along with calculating the empirical parameter "R, as discussed by Dahn et al.^10^," was carried out. "The ratio of the height of the (002) Bragg peak to the background defines the R parameter"^11^. A ratio of (B)/(A) delivers the value for the R parameter where "(A) is a straight line connecting the peaks on each side and (B) is a tangent to the linear background intersecting the (002) peak in a single point"^11^. The lateral crystallite size (L_a_), stacking crystallite height (L_c_), the average number of layers per carbon crystallite (N _average_), and the average number of carbon atoms (n) per aromatic lamellae were calculated based on Scherrer's equation.

1 $$ {\text{L}}_{{\text{a}}} = {\text{ K}}\lambda /\beta_{{\text{a}}} {\cos}\theta_{{\text{a}}} $$

2 $$ {\text{L}}_{{\text{c}}} = {\text{ K}}\lambda /\beta_{{\text{c}}} {\cos}\theta_{{\text{c}}} $$

3 $$ {\text{N}}_{{({\text{average}})}} = {1 } + {\text{ L}}_{{\text{c}}} /{\text{d}}_{{00{2}}} $$

4 $$ n = 0.32N^{2} $$

Table 2 clarifies that although SAC is of higher rank, the XRD structural analyses foretell that it has limited electrochemical storage in comparison with HVBC as the "R" value inferred. Furthermore, an increase in L_a_ and L_c_ further justifies the difference in the crystallite size between the two coals. As denoted in our previous work, an increase in L_a_ and L_c_ hinders proper electrochemical stocking^16^. Slightly higher N_(average)_ and n values were recorded for semi-anthracite coal along with an increase in the interlayer spacing (d_002_) that may suggest increased capacitive performance but not in all cases, a peculiar relationship between these two parameters was reported^23^.

**Table 2:** XRD structural analysis of high volatile bituminous and semi-anthracite coals.

Coal sample	L_a_	L_c_	d_002_	N	n	R
HVBC	36.47	20.86	3.47	6.8	15.7	2.3
SAC	40.2	21.4	3.50	7.1	16.1	2.8

**Figure 4:**
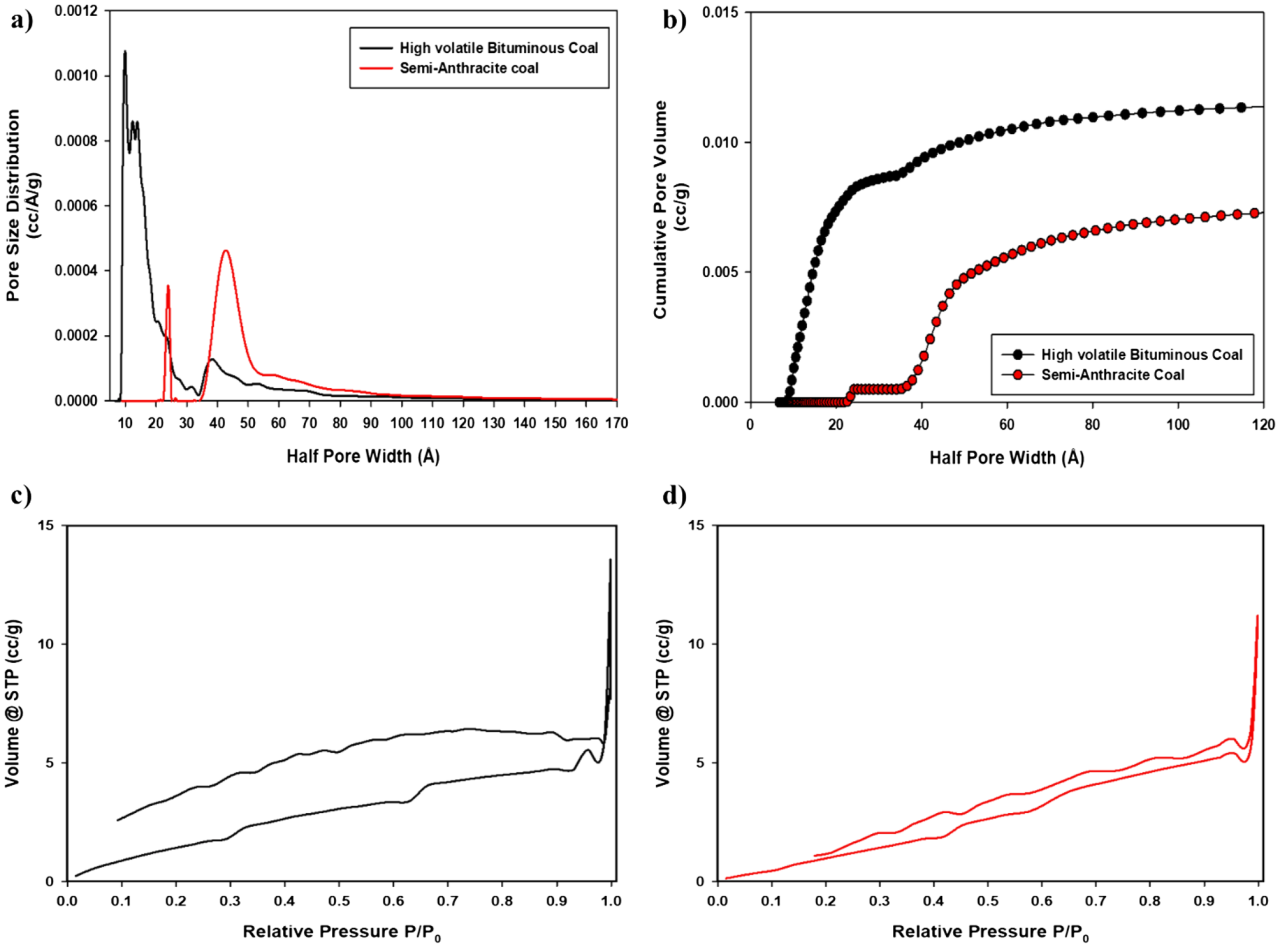
(**a**) Pore size distribution and (**b**) cumulative pore volume of high volatile bituminous (black) and semi-anthracite (red) coals. N_2_ adsorption isotherms of (**c**) HVBC and (**d**) SAC.

Characterizing the textural properties of both coal samples was made possible through the N_2_ adsorption method using a QUADRASORB EVO apparatus at 77 K. QSDFT models were used to obtain the pore size distribution (Fig. 4a), and the cumulative pore volume (Fig. 4b). The N_2_ adsorption–desorption isotherms of HVBC and SAC are displayed in Fig. 4c and d respectively. For both coal samples, the contribution of the microporous volume seems to be insignificant since the adsorption of N_2_ at low relative pressures (P/P_0_ < 0.10) is not very high. However, a strong N_2_ adsorption at high relative pressures (P/P_0_ > 0.95) was remarked, indicating the presence of mesoporous structures in these materials. As the isotherms indicate, the HVBC shows a higher hysteresis in comparison with SAC. For HVBC, it seems that some N_2_ molecules are trapped in the interlayer spacing, causing the hysteresis and explains the open end at low pressure. Notably, the pore size distribution in the micropores region was absent for both coals. High volatile bituminous coal displays two significant peaks at 4.07 and 5.95 nm. Meanwhile, semi-anthracite coal shows two significant peaks at bigger pores sizes, i.e., at 4.82 and 7.57 nm with lower pore size values (cc/nm/g) in comparison with HVBC. Furthermore, cumulative pore volume and specific surface area showed similar values. However, the pore size distribution for semi-anthracite coal was more prominent in the increased mesopores region. For facilitated comparison, data derived from QSDFT and BET models are summarized in Table 3.

**Table 3:** QSDFT and BET derived data concerning pore volume distribution as a function of size, cumulative pore volume, half pore width, and specific surface area of both coal samples.

Coal sample	V_M1_ (2–8 nm) (cc/g)	V_M2_ (> 8 nm)(cc/g)	V_M1_/V_M2_	C.P.V (cc/g)	P.W (nm)	S.S.A (m^2^/g)
HVBC	6.0 × 10^–3^	2.3 × 10^–3^	2.6	8.3 × 10^–3^	4.1	7.51
SAC	1.78 × 10^–3^	5.8 × 10^–3^	0.3	7.58 × 10^–3^	8.7	7.57

*C.P.V* Cumulative pore volume, *P.W* pore width, *S.S.A* specific surface area.

Remarkable distinctions were made regarding the porosity of the two coal samples. The small mesopores (< 8 nm) in the high volatile bituminous coal contributed to 72% of the overall cumulative volume. However, the decreased small mesopores evident in semi-anthracite coal (24% of the overall cumulative volume) may develop energetic hindrance in terms of decreased capacitive behavior. The central pore size distribution of the SAC is in the wider mesopores region (> 8 nm), registering 76% of the overall cumulative volume. Remarkably, the pore width of this coal was more than double (8.7 nm) that of HVBC (4.1 nm). Finally, the specific surface area is somehow similar, with minor augmentation in the semi-anthracite sample, as noted in previous sections, increasing pore width and decreased pore volume effect negatively on the energetic properties of the material.

**Figure 5:**
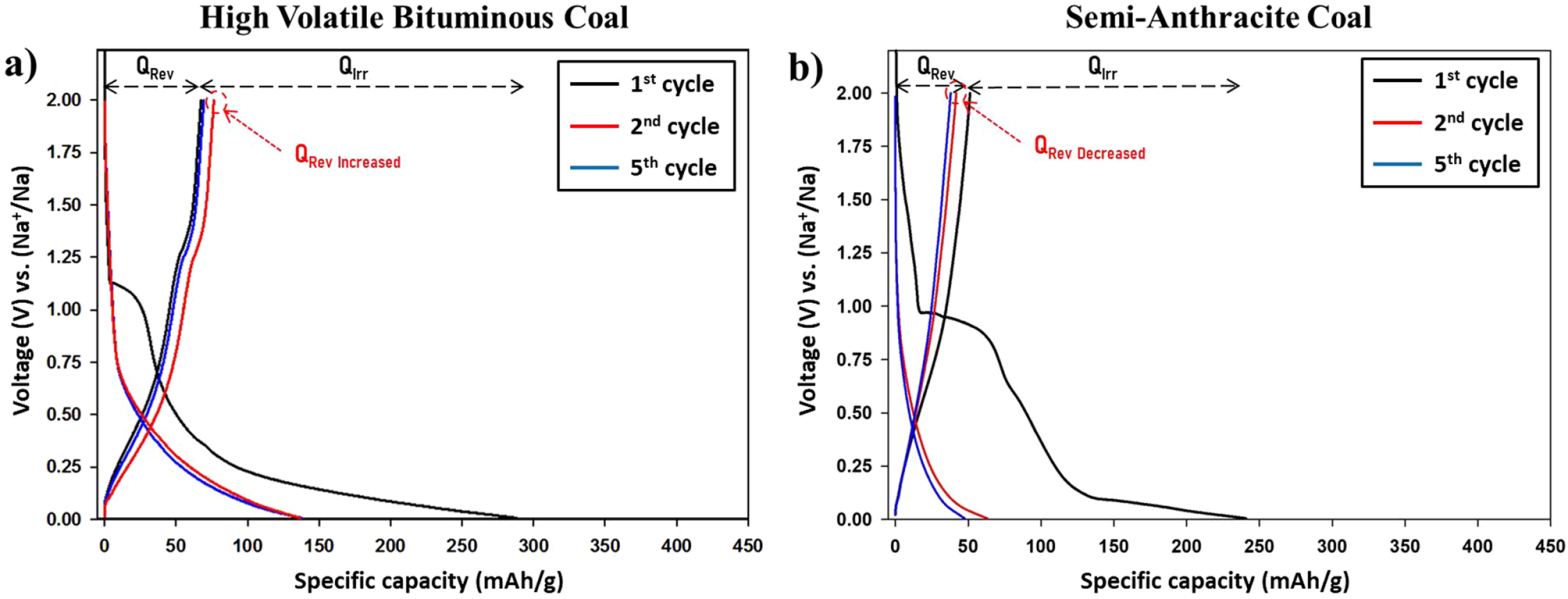
Galvanostatic profiles of (**a**) high volatile bituminous coal and (**b**) semi-anthracite coal in their raw states vs. Na with PC/FEC (95:5) at 12 mA/g.

Although SAC displayed a higher purity and enhanced structural ordering than HVBC, XRD structural analysis, and textural properties indicate an inferior electrochemical performance of this coal. Several electrodes series were tested, and different slurries were formulated. Nevertheless, the immense flake size of raw semi-anthracite and its poor homogeneity proved challenging to achieve repetitive results in NIBs. After several repetitions, representative results were attained for raw semi-anthracite represented in Fig. 5. Galvanostatic cycling of these materials further confirmed the annotations discussed previously. Primarily, the reduction reaction during the 1st cycle recorded a specific discharge capacity value of 287 mAh/g for HVBC and 245 mAh/g for SAC representing the electrolyte reduction along with sodiation reactions. Although the reduction profile attains similar shape, however, the reduction potentials are different where electrolyte reduction occurs at higher potentials in HVBC. The electrolyte reduction reaction was only recorded during the 1st cycle for both coals. The reduction slope occurring at 1 V displayed two distinct curve outlines. For HVBC, the sloping profile occurred between 1 V and ~ 0.35. In contrast, for SAC, this profile occurred between 1  and ~ 0.1 V, which is a representative of sodium adsorption to the surface of carbon layers. The second curve outline was a plateau taking place until 0.05 V, which is a representative of Na^+^ insertion into the micro-voids and spaces of the carbonaceous materials. In comparison with HVBC, this sodiation into the nano-spaces was less marked in SAC due to the altered textural aspects (as the R values suggested).

Meanwhile, during the charge cycle, both coals displayed similar profiles where a single slope representing desodiation reactions recorded a specific capacity of 67 mAh/g for HVBC and 51 mAh/g for SAC. The irreversible capacity during the 1st cycle was 220 mAh/g for HVBC and 194 mAh/g for SAC. During the 2nd cycle, the low potential plateaus were absent, further confirming the occupation of the nano-voids and spaces with Na^+^ and their inability to be removed during oxidation reactions. The reversible capacity during the second cycle increased in HVBC but decreased in SAC samples. However, during the 5th cycles, the reversible capacity of HVBC retained the initial capacity, i.e., 67 mAh/g, whereas SAC reversible capacity values further diminished to 34 mAh/g. Thus, in galvanostatic cycling, the reversible capacity was initially lower for SAC than that of the lower rank coal (HVBC), and the capacity retention was limited as the textural properties suggested. Thus, the impact of the coal rank on electrochemical storage of Na^+^ was noticed as the physico-chemical tests inferred.

Cyclic voltammetry for HVBC was reported and extensively discussed in our previous work^16^. In general, both coals showed electrolyte reduction and the SEI formation reactions during the 1st discharge (sodiation) reaction with peaks ~ 1 V attributed to the one-electron reductive process of this additive similar to other carbonaceous materials^24^. Reversible sodiation reactions occur between 1  and ~ 0 V. Oxidation peaks resembling desodiation were recorded at 0.1 V. These coals display similar curve shapes and behaviors as other reported carbonaceous materials^25,26^. However, the difference in reversible currents between the coals was recorded. The voltammogram of SAC displayed a sodiation peak with lower currents than HVBC (12 mA/g). Nevertheless, the oxidation reaction during the 1st cycle recorded low values (~ 0 mA/g) hence displaying increased irreversibility of sodium extraction during the discharge cycle in SAC. These results were expected since the material showed poor physico-chemical properties (R values, d_002_, and porosity). During the 2nd and 5th cycles, reduced currents were recorded throughout the reduction reactions, further demonstrating the inferior Na^+^ intercalation capability of SAC. Thus, raw SAC fails to display reversible cycling with poor electrochemical in comparison with HVBC, where it shows inferior performance and cyclability.

In our previous work, the effect of low-temperature thermal treatment on HVBC was discussed. The electrochemical storage behavior and performance of these thermally treated samples were immensely enhanced. The reversible capacities recording of the thermally processed HVBC at 800 °C was threefold that of the raw HVBC. Hence, to further investigate the impact of coal rank in electrochemical storage of Na^+^, similar thermal pyrolysis processes were carried out on SAC with active product cycled in similar conditions as thermally modified HVBC. Nonetheless, the effect of thermal treatment on SAC coal was not the same. Physico-chemical characterization displayed the effect of thermal treatment on the structure, composition, and textural properties with similar conclusions as in HVBC. Thermal annealing at 800 °C under argon showed that heat treatment has does not affect the distribution of impurities; certain homogeneity in the size of the grains was noticed as a direct effect of thermal treatment; pyrite transformation into iron oxides; changes in the structure after thermal treatment. The structural changes were detected via Raman and XRD analyses, where augmentation in the intensity of the D band was detected. Unlike graphite, the increase of the I(D)/I(G) ratio (i.e., the development of the D band) designated increased ordering^22^. Increased ordering for HVBC was more pronounced than SAC due to its lower rank. The I_D_/I_G_ ratio for thermally treated HVBC was 0.86, whereas for SAC was 0.72 indicating the decreased ordering of SAC after thermal treatment than HVBC. The bigger flake size of SAC could be a factor in the previous observation.

Meanwhile, in XRD, both coals detected the transformation of pyrite into hematite with its hkl recordings (104 and 110). Additional XRD structural analyses were carried out to determine the effect of thermal treatment on the crystalline lateral and stacking size, interlayer spacing, an average number of layers per carbon crystallite, carbon atoms per aromatic lamellae and R values as displayed in Table 4. Thermal treatment decreases the lateral crystalline size, increases the interlayer spacing and the R-value of the sample. All these parameters predict the enhanced electrochemical performance of thermally treated coals as active anode materials.

**Table 4:** XRD structural analysis of HVBC and SAC after thermal treatment at 800 °C under argon.

Coal sample	L_(a)_	L_(c)_	d_002_	N_(average)_	n	R
HVBC 800 °C	28	23	3.55	7.5	18.0	1.8
SAC 800 °C	32.5	20.4	3.59	6.7	14.3	2.3

**Figure 6:**
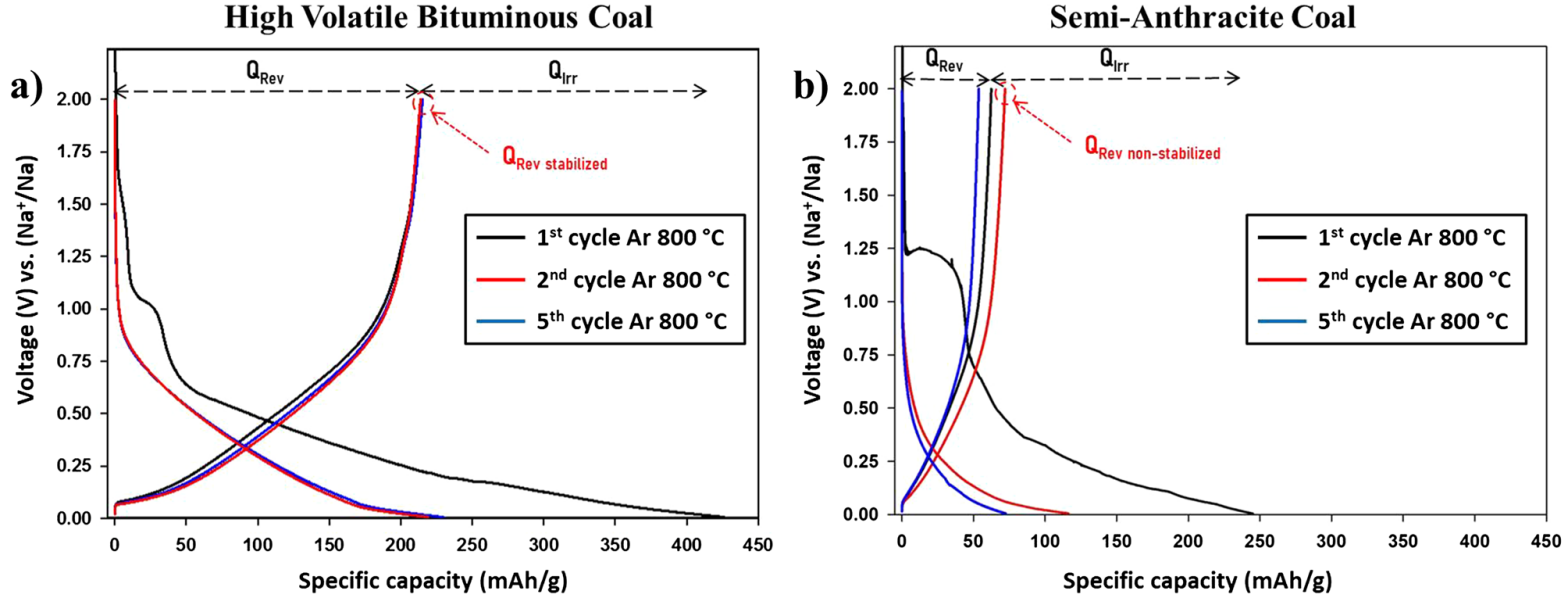
Galvanostatic profiles of (**a**) high volatile bituminous coal and (**b**) semi-anthracite coal after pyrolysis at 800 °C under argon vs. Na with PC/FEC (95:5) at 12 mA/g.

Changes in terms of electrochemical performance were recorded, as displayed in Fig. 6. Distinct galvanostatic profiles were recorded for the samples after thermal processing at 800 °C under argon. As noticed in raw coals, the 1st discharge (sodiation) cycle demonstrates electrolyte reduction with SEI formulation and sodiation reaction taking place in the nano-voids and spaces. The sloping curves are different between the sample where HVBC displays electrolyte reduction at lower potentials ~ 1.0 V. In contrast, SAC commences these reactions at ~ 1.2 V. The recorded discharge capacity of the former coal was 426 mAh/g, whereas the latter was 245 mAh/g. The difference in this capacity is due to the increased occupied spaces by Na^+^ rather than the non-reversible electrolyte reduction and SEI formation. This was evident since the reversible specific capacity during the charge reaction of thermally treated HVBC was 214 mAh/g, whereas SAC was 64 mAh/g. During the 2nd and 5th, cycles thermally treated HVBC attains the same reversible capacity as the 1st cycle, whereas SAC shows an increase in the reversible capacity to 72 mAh/g followed by a decrease to 53 mAh/g during the 2nd and 5th cycle, respectively. The reversible capacity changes were still occurring in SAC even after thermal pyrolysis, whereas it was restrained in HVBC. In comparison with the thermally treated HVBC, the capacities recorded were minute since the reversible capacity recorded was almost threefold lower than SAC, i.e., almost the same capacity as raw HVBC.

**Figure 7:**
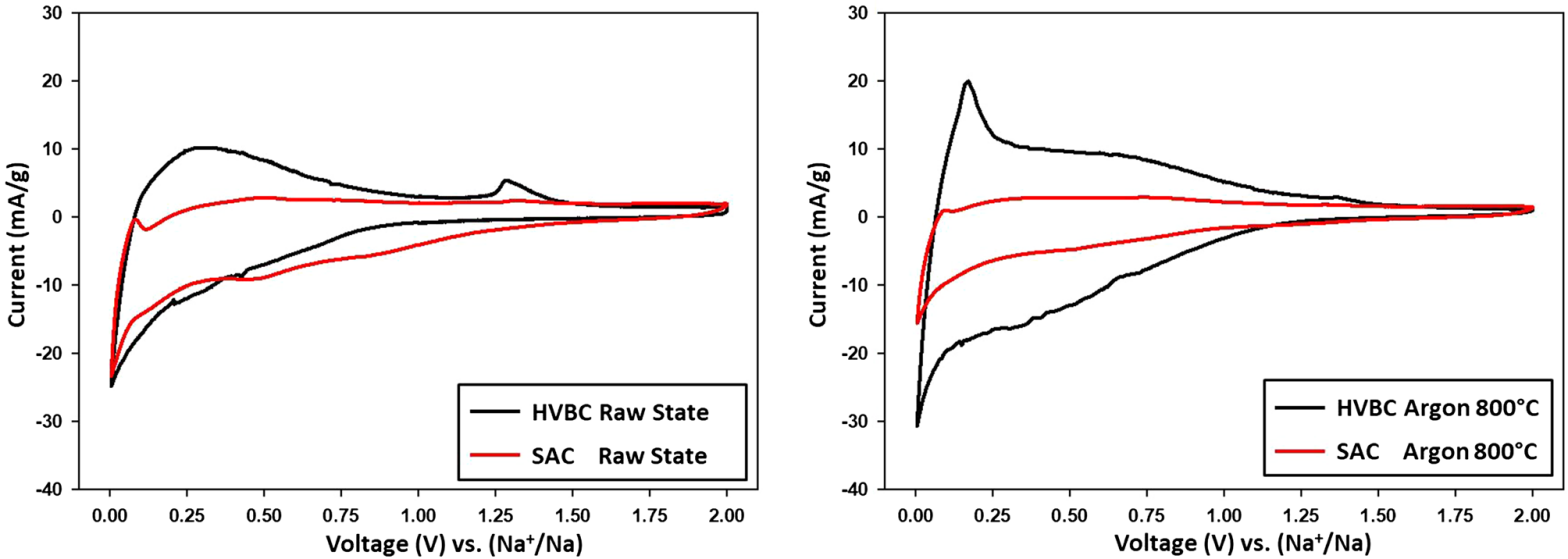
Cyclic voltammetry curves at 25 μV vs. Na with PC/FEC (95:5) of raw coals (left) and thermally treated coals (right). High volatile bituminous coal is represented in red, and semi-anthracite coal is represented in black.

Moreover, the cyclic voltammograms after electrode reduction (i.e., after the first cycle) were distinctly different since the storage capacity presented by the oxidation area, and the intensity of the peak of the de-sodiation reaction (visible at 0.18 mV) was more pronounced in the HVBC coal after thermal treatment with an oxidation peak recording 20 mA/g (Fig. 7). In contrast, SAC displayed slightly enhanced peak shape with minute recordings. Finally, the more prominent peaks after thermal treatment having elevated currents, suggests an increased capacitive storage capability along with increased reversibility of Na^+^ intercalation/deintercalation for HVBC in comparison with SAC in coherence with previous GCPL observations where the reversible specific capacity was higher for the prior coal.

## Conclusion

As Dahn et al. reported, the empirical value calculated from XRD can foresee the energy storage capability of coals in LIBs as it can also provide the same information in NIBs. In this work, we further verified this observation along with displaying a similar proposition for coal-based carbon anodes in NIBs. Furthermore, textural analysis displaying the pore volume can further suggest similar energetic propositions. The absence of micropores will impact the energetic properties in coals. This matter was also verified when thermal treatment was applied to high volatile bituminous coal and semi-anthracite coal. The textural properties of the latter were inadequate in comparison with the prior coal, although this it has decreased ash and volatile matter content. After thermal treatment, HVBC showed enhanced electrochemical performance due to the increase of microporosity/other textural and structural properties (R-value) than SAC. The impact of the coal rank on the electrochemical performance was clear since HVBC displayed a reversible capacity of 67 mAh/g in its raw state and 214 mAh/g after pyrolysis at 800 °C under argon vs. Na. On the other hand, SAC recorded a reversible capacity of 51 mAh/g in its raw state and 64 mAh/g after pyrolysis at 800 °C under argon vs. Na. It was further suggested that the flake size of the coal further impacts the energetic performance of coals. The semi-anthracite, although higher in rank than high volatile bituminous coal, displayed large flake size and bigger pore width, which translated into high overall irreversible capacity.

As a conclusion, it was observed that impurity presence (ash content) and volatile matter content were of minor effect on the energetic properties of natural coals. The structural parameters in terms of textural properties are prominent contributors. Furthermore, thermal treatment was a valuable tool to enhance the energetic possibilities of natural coals regardless of their rank. However, some coals are more responsive to these thermal processes and display higher energetic activity due to the enhanced structural and textural properties. To complete the study regarding the impact of coal rank on the energetic properties in NIBs, we suggest assessing natural lignite coal with similar experiments.

## Data Availability

The datasets generated during and/or analyzed during the current study are available from the corresponding author on reasonable request.
